# Supplementary material for: Identifying Effects of Urinary Metals on Type 2 Diabetes in U.S. Adults: Cross-Sectional Analysis of National Health and Nutrition Examination Survey 2011–2016
Source: Nutrients. 2022 Apr 8;14(8):1552. doi: 10.3390/nu14081552 (PMC9031490; doi:10.3390/nu14081552)
Supplement: Supplementary file 1 [file nutrients-14-01552-s001.zip › nutrients-1591269-supplementary.pdf]

Identifying effects of urinary metals on type 2 diabetes in U.S. adults: Cross-sectional analysis of National Health and Nutrition Examination Survey 2011–2016

Supplementary Materials

Figure S1. Flowchart of participants selection. 2

Figure S2. Study design and analysis process 3

Table S1. The lower limit of detection (LOD, in μg/L) for the urinary metals 4

Table S2. Geometric mean of urinary metals levels 5

Figure S3. Elastic net analysis on the association of metal mixtures and type 2 diabetes 6

Table S3. Adjusted odds ratio for prevalence of type 2 diabetes by urinary metal levels 7

Figure S4. Univariate exposure-response functions and 95% confidence intervals (shaded areas) for each metal with the other metals holding at the median. 8

Figure S5. The interaction of urinary metals on type 2 diabetes. 9

Table S4. Adjusted odds ratio for type 2 diabetes according to Sn and Sr levels 10

Figure S6. Overall effect of the mixture estimates and 95% confidence interval. 11

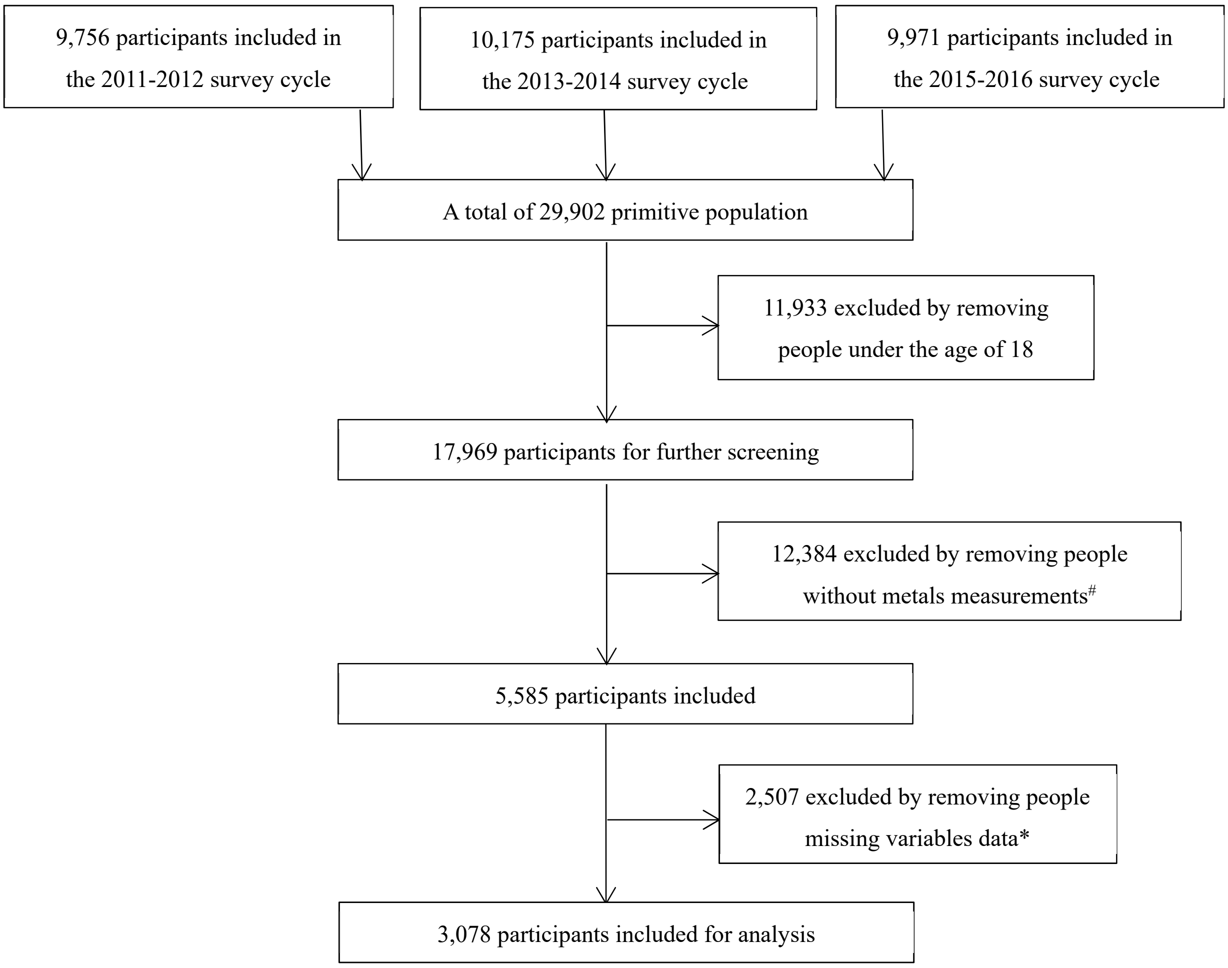

Figure S1. Flowchart of participants selection.

12357 excluded removing people without Ba, Cd, Co, Mn, Pb, Sb, TL, or U measurements, 12358 without Cs, Mo, Sr, Sn, W, or As measurements, 12346 without Hg measurements.

* 291 excluded by removing people missing educational attainment data, 285 missing poverty income ratio data, 98 missing smoking status data, 522 missing alcohol consumption data, 65 missing body mass index data, 349 missing average daily energy intake data, 2091 missing family history of diabetes data, 241 missing hypertension data, 206 missing alanine aminotransferase data, 205 missing gamma-glutamyl transferase data, 339 missing estimated glomerular filtration rate data.

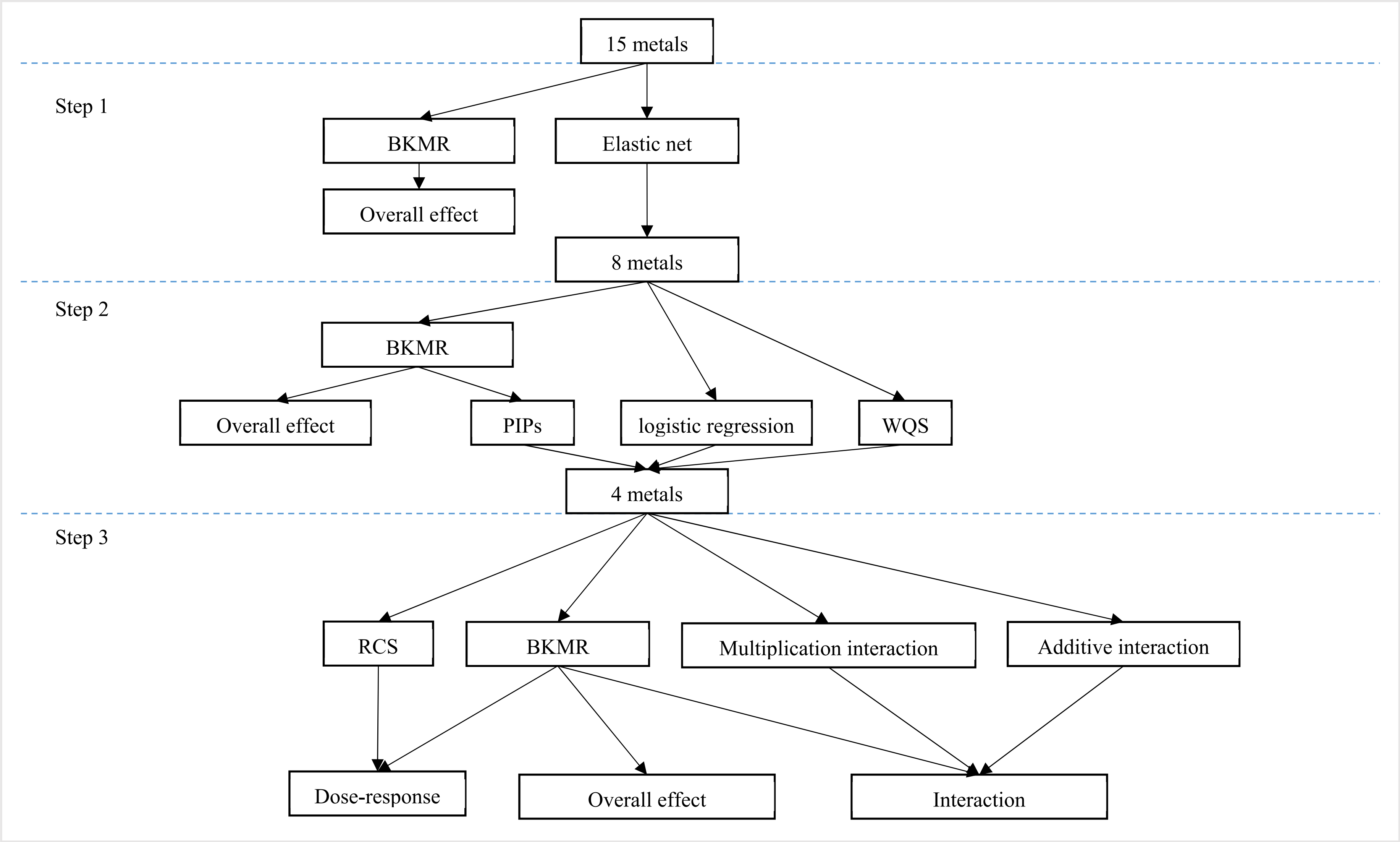

Figure S2. Study design and analysis process

Abbreviations: BKMR, Bayesian kernel machine regression model; PIPs, Posterior inclusion probabilities; RCS, restricted cubic spline; WQS, Weighted quantile sum regression.

Table S1. The lower limit of detection (LOD, in μg/L) for the urinary metals

Variable Name	LOD, in μg/L	Below lower detection limit, %
Ba	0.060	0.6
Cd	0.036	9.6
Cs	0.086	0.0
Co	0.023	0.2
Mn	0.130	61.6
Mo	0.800	0.0
Pb	0.030	1.7
Sb	0.022	28.8
Sr	2.340	0.0
TL	0.018	0.6
Sn	0.090	11.3
W	0.018	18.6
U	0.002	21.6
Hg	0.130	25.3
As	0.260	1.0

Table S2. Geometric mean of urinary metals levels

	Without T2D (n=2,583)	With T2D (n=495)	Total people (n=3,078)	*P* values
Antimony (Sb), 10^{-2} μg/g of Cr				<0.001
Mean (SD)	0.711 (0.299)	0.723 (0.285)	0.713 (0.296)	
Median [Min, Max]	0.684 [-0.0677, 2.26]	0.696 [-0.0388, 2.06]	0.686 [-0.0677, 2.26]	
Arsenic (As), μg/g of Cr				<0.001
Mean (SD)	0.915 (0.455)	0.926 (0.411)	0.916 (0.449)	
Median [Min, Max]	0.831 [-0.261, 2.86]	0.876 [-0.0378, 2.20]	0.836 [-0.261, 2.86]	
Barium (Ba), 10^{-1} μg/g of Cr				<0.001
Mean (SD)	1.06 (0.399)	1.00 (0.464)	1.05 (0.411)	
Median [Min, Max]	1.06 [-0.553, 2.64]	1.01 [-0.757, 2.54]	1.05 [-0.757, 2.64]	
Cadmium (Cd), 10^{-2} μg/g of Cr				<0.001
Mean (SD)	1.27 (0.396)	1.42 (0.351)	1.29 (0.393)	
Median [Min, Max]	1.27 [-0.226, 2.88]	1.42 [0.262, 2.56]	1.30 [-0.226, 2.88]	
Cesium (Cs), 10^{-1} μg/g of Cr				<0.001
Mean (SD)	1.62 (0.220)	1.62 (0.204)	1.62 (0.217)	
Median [Min, Max]	1.61 [-0.419, 2.63]	1.64 [0.834, 2.26]	1.62 [-0.419, 2.63]	
Cobalt (Co), 10^{-2} μg/g of Cr				<0.001
Mean (SD)	1.57 (0.282)	1.59 (0.307)	1.57 (0.286)	
Median [Min, Max]	1.55 [0.574, 3.32]	1.55 [0.414, 3.54]	1.55 [0.414, 3.54]	
Lead (Pb), 10^{-2} μg/g of Cr				<0.001
Mean (SD)	1.56 (0.322)	1.60 (0.309)	1.57 (0.321)	
Median [Min, Max]	1.55 [0.561, 3.35]	1.60 [0.768, 3.15]	1.56 [0.561, 3.35]	
Manganese (Mn), 10^{-2} μg/g of Cr				<0.001
Mean (SD)	1.09 (0.354)	1.06 (0.304)	1.09 (0.347)	
Median [Min, Max]	1.06 [0.0737, 3.12]	1.02 [0.286, 2.36]	1.05 [0.0737, 3.12]	
Mercury (Hg), 10^{-2} μg/g of Cr				<0.001
Mean (SD)	1.47 (0.446)	1.45 (0.437)	1.46 (0.444)	
Median [Min, Max]	1.45 [0.301, 3.56]	1.43 [0.291, 3.76]	1.45 [0.291, 3.76]	
Molybdenum (Mo), μg/g of Cr				<0.001
Mean (SD)	1.54 (0.279)	1.57 (0.278)	1.55 (0.278)	
Median [Min, Max]	1.55 [0.413, 2.50]	1.59 [0.612, 2.63]	1.55 [0.413, 2.63	
Strontium (Sr), 10 μg/g of Cr				<0.001
Mean (SD)	0.967 (0.317)	0.906 (0.373)	0.957 (0.328)	
Median [Min, Max]	0.982 [-0.860, 2.27]	0.955 [-0.868, 1.72]	0.979 [-0.868, 2.27]	
Thallium (TL), 10^{-2} μg/g of Cr				<0.001
Mean (SD)	1.20 (0.243)	1.18 (0.253)	1.20 (0.245)	
Median [Min, Max]	1.20 [-0.156, 2.32]	1.17 [0.269, 2.48]	1.19 [-0.156, 2.48]	
Tin (Sn), 10^{-1} μg/g of Cr				<0.001
Mean (SD)	0.691 (0.431)	0.838 (0.450)	0.715 (0.437)	
Median [Min, Max]	0.637 [-0.376, 2.83]	0.771 [-0.231, 2.64]	0.660 [-0.376, 2.83]	
Tungsten (W), 10^{-2} μg/g of Cr				<0.001
Mean (SD)	0.799 (0.381)	0.800 (0.368)	0.799 (0.378)	
Median [Min, Max]	0.773 [-0.258, 2.99]	0.792 [-0.212, 2.67]	0.776 [-0.258, 2.99]	
Uranium (U), 10^{-3} μg/g of Cr				<0.001
Mean (SD)	0.760 (0.390)	0.809 (0.398)	0.768 (0.392)	
Median [Min, Max]	0.716 [-0.341, 3.01]	0.754 [-0.00616, 2.49]	0.721 [-0.341, 3.01]	

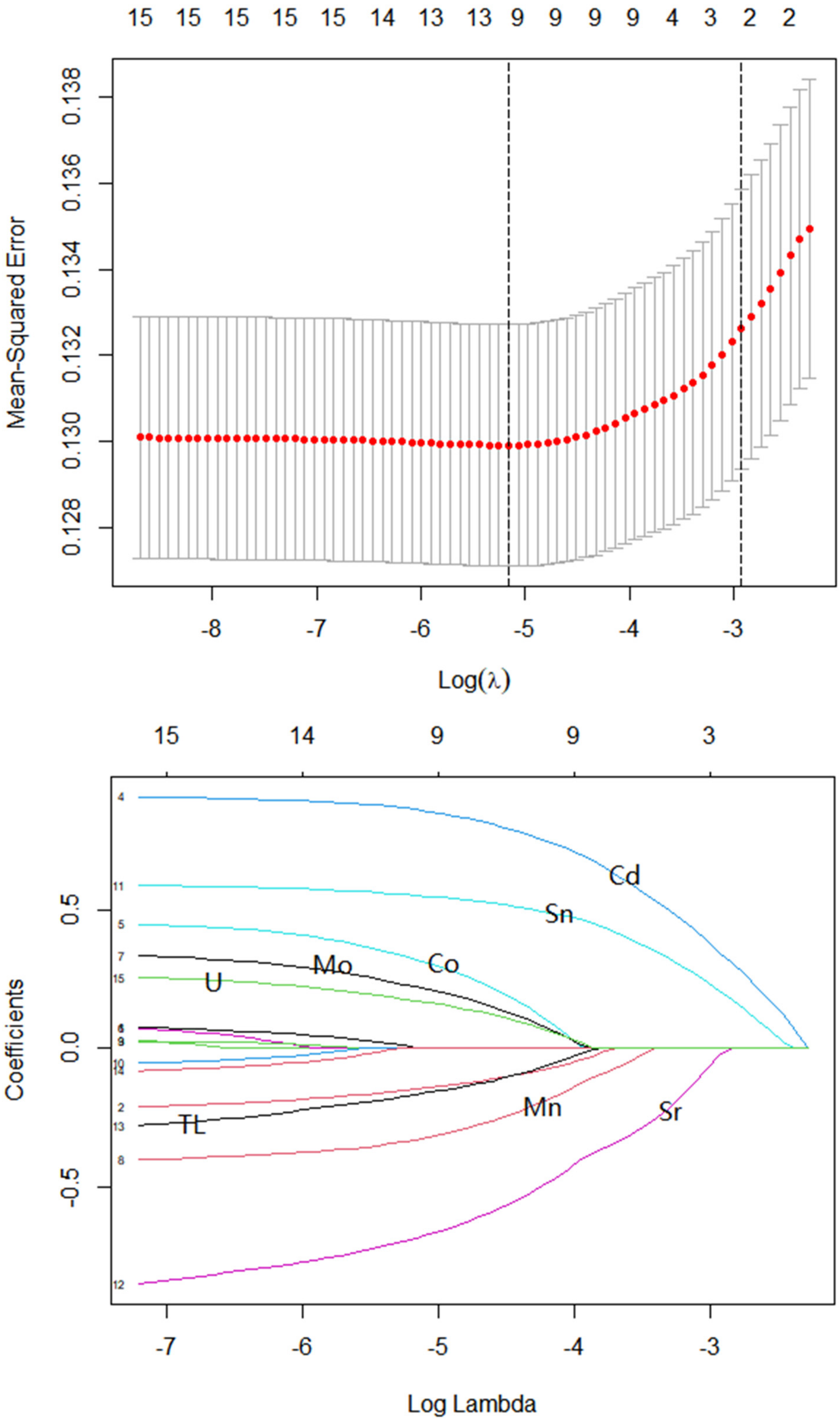

Figure S3. Elastic net analysis on the association of metal mixtures and type 2 diabetes

Abbreviations: As, Arsenic; Cd, Cadmium; Co, Cobalt; Mn, Manganese; Mo, Molybdenum; Sn, Tin; Sr, Strontium; TL, Thallium.

Table S3. Adjusted odds ratio for prevalence of type 2 diabetes by urinary metal levels

Variables	Quartiles of urinary metals (10^{-2} μg/g of creatinine)				P_{trend} [a]	Linear model
	Quantile 1	Quantile 2	Quantile 3	Quantile 4		
Cadmium (Cd)	≤ 10.55	10.56–19.72	19.73–36.06	≥ 36.07	---	---
N (case/total)	60/770	113/769	153/770	169/769	---	495/3,078
Crude [b]	1.00 (reference)	2.19 (1.42, 3.40)	3.13 (1.99, 4.91)	3.61 (2.33, 5.61)	<0.01	2.88 (2.11, 3.93)
Model 1 [c]	1.00 (reference)	1.44 (0.89, 2.33)	1.68 (0.96, 2.93)	2.00 (1.15, 3.48)	0.02	1.79 (1.06, 3.01)
Model 2 [d]	1.00 (reference)	0.71 (0.35, 1.42)	1.20 (0.62, 2.34)	1.03 (0.46, 1.39)	0.17	1.56 (0.91, 2.67)
Cobalt (Co)	≤ 23.94	23.98–35.14	35.14–53.72	≥ 53.75	---	---
N (case/total)	122/770	123/769	114/770	136/769	---	495/3,078
Crude [b]	1.00 (reference)	0.81 (0.60, 1.08)	1.05 (0.74, 1.49)	1.05 (0.80, 1.37)	0.38	1.40 (0.89, 2.20)
Model 1 [c]	1.00 (reference)	0.75 (0.54, 1.05)	1.27 (0.83, 1.95)	1.12 (0.74, 1.68)	0.26	1.48 (0.87, 2.52)
Model 2 [d]	1.00 (reference)	1.17 (0.73, 1.86)	1.21 (0.77, 1.88)	1.63 (0.96, 2.79)	0.13	1.83 (1.00, 3.34)
Manganese (Mn)	≤ 6.97	6.97–11.28	11.30–20.00	≥ 20.00	---	---
N (case/total)	115/770	148/769	121/770	111/769	---	495/3,078
Crude [b]	1.00 (reference)	1.55 (1.10, 2.18)	1.23 (0.86, 1.76)	1.02 (0.73, 1.42)	0.54	0.76 (0.56, 1.03)
Model 1 [c]	1.00 (reference)	1.87 (1.23, 2.86)	1.40 (0.89, 2.20)	1.26 (0.79, 2.00)	0.73	0.87 (0.55, 1.37)
Model 2 [d]	1.00 (reference)	1.90 (1.12, 2.59)	1.33 (0.88, 2.01)	1.10 (0.71, 1.69)	0.84	0.72 (0.45, 1.16)
Molybdenum (Mo)	≤ 2379.30	2379.00–3582.00	3589.00–5252.00	≥ 5255.00	---	---
N (case/total)	109/770	104/769	148/770	134/769	---	495/3,078
Crude [b]	1.00 (reference)	0.72 (0.49, 1.06)	1.39 (0.95, 2.01)	1.23 (0.96, 1.58)	0.01	1.42 (0.98, 2.06)
Model 1 [c]	1.00 (reference)	0.73 (0.49, 1.09)	1.40 (0.90, 2.17)	1.37 (1.00, 1.88)	0.01	1.59 (1.03, 2.45)
Model 2 [d]	1.00 (reference)	0.95 (0.66, 1.39)	1.66 (1.09, 2.51)	1.18 (0.84, 1.67)	0.10	1.44 (0.96, 2.17)
Strontium (Sr)	≤ 5889.90	5897.00–9525	9529–146644	≥ 14660	---	---
N (case/total)	151/770	110/769	120/770	114/769	---	495/3,078
Crude [b]	1.00 (reference)	0.66 (0.46, 0.96)	0.73 (0.48, 1.12)	0.80 (0.61, 1.05)	0.17	0.59 (0.43, 0.82)
Model 1 [c]	1.00 (reference)	0.64 (0.45, 0.91)	0.75 (0.46, 1.21)	0.75 (0.52, 1.08)	0.22	0.58 (0.38, 0.87)
Model 2 [d]	1.00 (reference)	0.57 (0.38, 0.85)	0.63 (0.36, 1.12)	0.53 (0.32, 0.88)	0.12	0.39 (0.23, 0.64)
Thallium (TL)	≤ 11.00	11.00–15.60	15.61–22.32	≥ 22.32	---	---
N (case/total)	142/770	128/769	107/770	118/769	---	495/3,078
Crude [b]	1.00 (reference)	0.81 (0.52, 1.27)	0.64 (0.41, 1.01)	0.90 (0.58, 1.40)	0.58	0.75 (0.39, 1.45)
Model 1 [c]	1.00 (reference)	0.92 (0.53, 1.57)	0.71 (0.42, 1.19)	1.24 (0.75, 2.05)	0.53	1.20 (0.55, 2.59)
Model 2 [d]	1.00 (reference)	0.77 (0.47, 1.25)	0.76 (0.45, 1.27)	1.14 (0.71, 1.84)	0.68	1.11 (0.49, 2.40)
Tin (Sn)	≤ 25.81	25.86–45.65	45.71–91.67	≥ 91.77	---	---
N (case/total)	81/770	107/769	135/770	172/769	---	495/3,078
Crude [b]	1.00 (reference)	1.15 (0.75, 1.76)	1.81 (1.10, 2.95)	2.29 (1.46, 3.58)	<0.01	2.10 (1.51, 2.94)
Model 1 [c]	1.00 (reference)	1.08 (0.68, 1.72)	1.65 (0.90, 3.02)	1.79 (1.07, 2.98)	0.02	1.65 (1.15, 2.36)
Model 2 [d]	1.00 (reference)	1.12 (0.70, 1.80)	1.42 (0.87, 2.30)	1.45 (0.83, 2.54)	0.12	1.53 (1.11, 2.20)
Uranium (U)	≤ 0.32	0.32–0.53	0.53–0.97	≥ 0.97	---	---
N (case/total)	101/770	131/769	130/770	133/769	---	495/3,078
Crude [b]	1.00 (reference)	1.59 (1.02, 2.49)	1.57 (1.01, 2.45)	1.66 (1.22, 2.27)	<0.01	1.39 (1.13, 1.71)
Model 1 [c]	1.00 (reference)	1.62 (0.96, 3.73)	1.49 (0.88, 2.53)	1.55 (1.00, 2.40)	0.09	1.32 (0.97, 1.78)
Model 2 [d]	1.00 (reference)	1.46 (0.89, 2.38)	1.33 (0.80, 2.19)	1.48 (0.97, 2.27)	0.13	1.39 (1.03, 1.88)

[a] P_{trend} across quartiles of metals was obtained by including the median of each quartile (log-transformed metals concentration) as a continuous variable. [b] Metals were included in the unadjusted unconditional logistic regression models separately. [c] Model 1 adjusted for sex, age, race/ethnicity, educational attainment, poverty income ratio, smoking status, alcohol consumption, physical activity, body mass index, average daily energy intake, family history of diabetes, hypertension, alanine aminotransferase, gamma-glutamyl transferase, and estimated glomerular filtration rate. [d] Model 2 further adjusted for other metals.

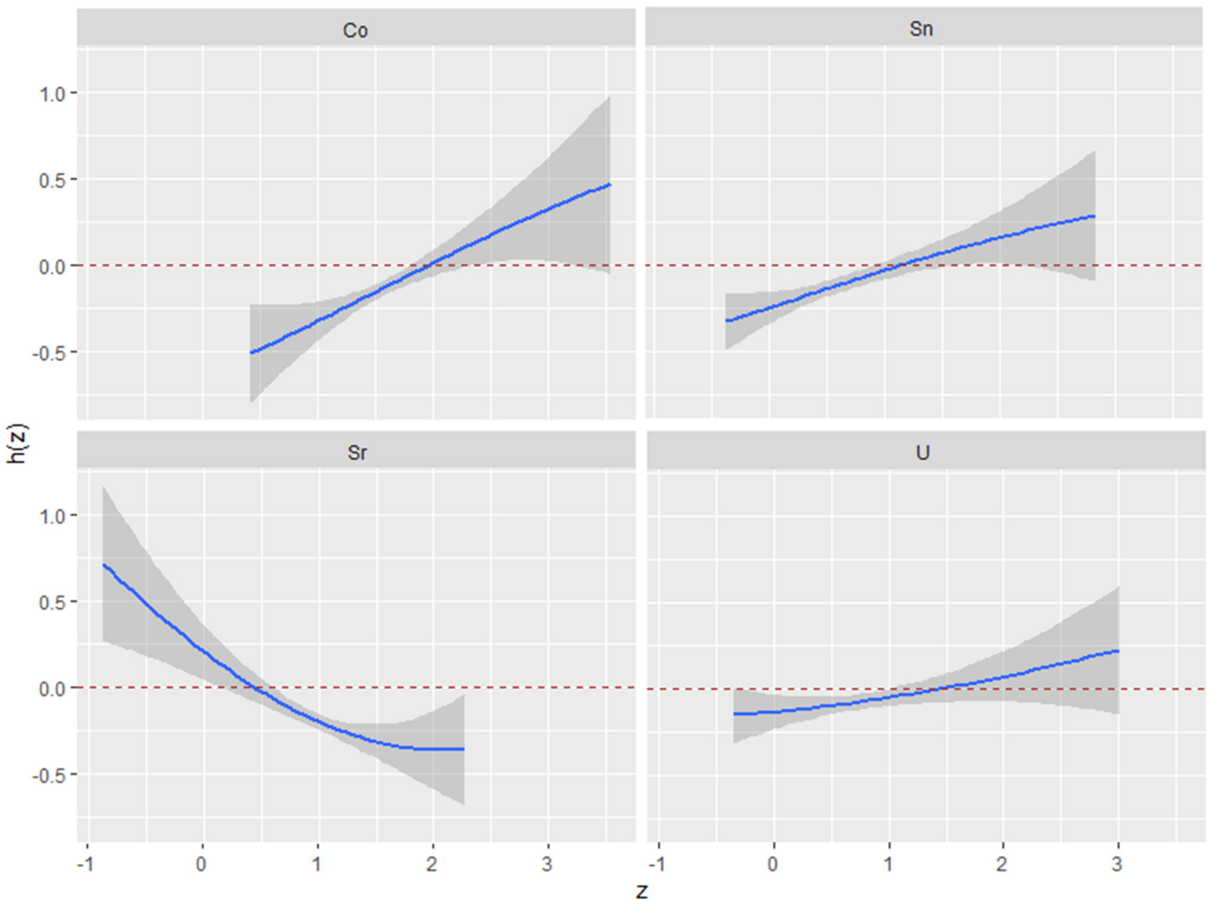

Figure S4. Univariate exposure-response functions and 95% confidence intervals (shaded areas) for each metal with the other metals holding at the median.

Abbreviations: Co, Cobalt; Sn, Tin; Sr, Strontium; U, Uranium. Data were estimated by Bayesian Kernel Machine Regression, while adjusting for sex, age, race/ethnicity, educational attainment, poverty income ratio, smoking status, alcohol consumption, physical activity, body mass index, average daily energy intake, family history of diabetes, hypertension, alanine aminotransferase, gamma-glutamyl transferase, and estimated glomerular filtration rate, and urinary cobalt, tin, strontium, uranium.

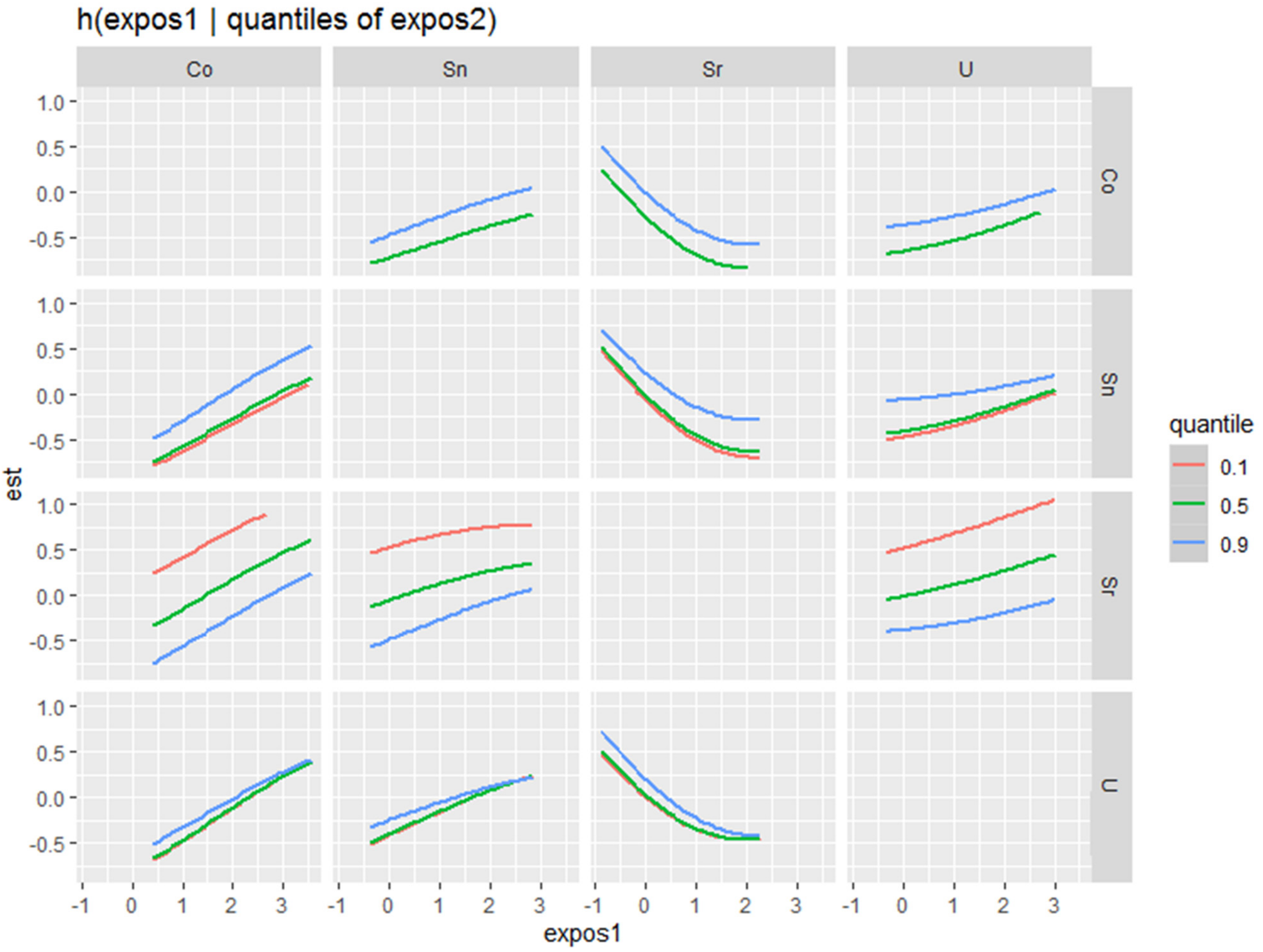

Figure S5. The interaction of urinary metals on type 2 diabetes.

Abbreviations: Co, Cobalt; Sn, Tin; Sr, Strontium; U, Uranium. Data were estimated by Bayesian Kernel Machine Regression, while adjusting for sex, age, race/ethnicity, educational attainment, poverty income ratio, smoking status, alcohol consumption, physical activity, body mass index, average daily energy intake, family history of diabetes, hypertension, alanine aminotransferase, gamma-glutamyl transferase, and estimated glomerular filtration rate, and urinary cobalt, tin, strontium, uranium.

Table S4. Adjusted odds ratio for type 2 diabetes according to Sn and Sr levels

		Sn, 10^{-2} ug/g of Cr	
		≥4.57	<4.57
Sr, 10^{-2} ug/g of Cr	<9.53	1.00 (reference)	0.98 (0.72, 1.33)
	≥9.53	1.19 (0.89, 1.60)	0.65 (0.45, 0.92)

Abbreviations: Sn, Tin; Sr, Strontium. Model adjusted for Model adjusted for sex, age, race/ethnicity, educational attainment, poverty income ratio, smoking status, alcohol consumption, physical activity, body mass index, average daily energy intake, family history of diabetes, hypertension, alanine aminotransferase, gamma-glutamyl transferase, and estimated glomerular filtration rate.

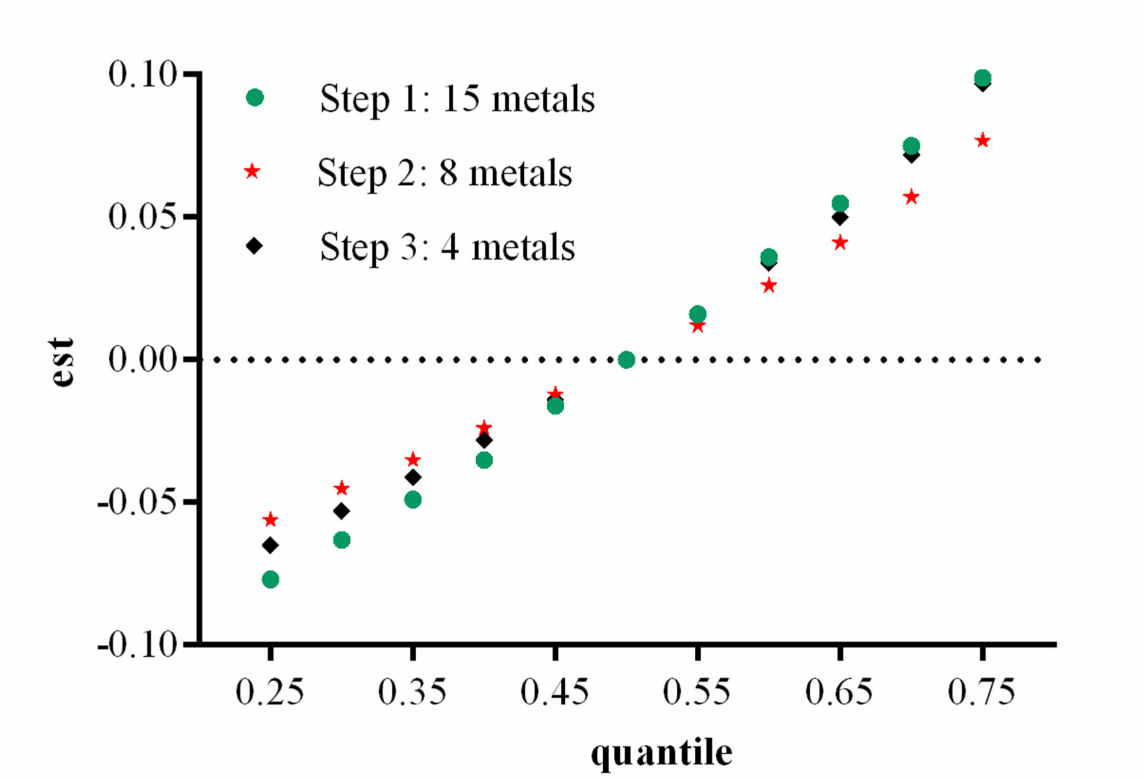

Quantile	0.25	0.30	0.35	0.40	0.45	0.50	0.55	0.60	0.65	0.70	0.75
**Step 1**											
est	-0.077	-0.063	-0.049	-0.035	-0.016	0.000	0.016	0.036	0.055	0.075	0.099
95% CI	[-0.139, -0.015]	[-0.108, -0.017]	[-0.081, -0.017]	[-0.055, -0.015]	[-0.026, -0.006]	[0.000, 0.000]	[0.006, 0.025]	[0.016, 0.056]	[0.025, 0.086]	[0.032, 0.118]	[0.040, 0.158]
**Step 2**											
est	-0.056	-0.045	-0.035	-0.024	-0.012	0.000	0.012	0.026	0.041	0.057	0.077
95% CI	[-0.092, -0.019]	[-0.073, -0.017]	[-0.055, -0.014]	[-0.038, -0.010]	[-0.019, -0.005]	[0.000, 0.000]	[0.005, 0.019]	[0.012, 0.040]	[0.020, 0.063]	[0.028, 0.086]	[0.038, 0.115]
**Step 3**											
est	-0.065	-0.053	-0.041	-0.028	-0.014	0.000	0.015	0.034	0.050	0.072	0.097
95% CI	[-0.106, -0.024]	[-0.083, -0.022]	[-0.063, -0.020]	[-0.042, -0.014]	[-0.021, -0.008]	[0.000, 0.000]	[0.008, 0.022]	[0.020, 0.048]	[0.028, 0.072]	[0.042, 0.102]	[0.057, 0.138]

Figure S6. Overall effect of the mixture estimates and 95% confidence interval.

Data were estimated by Bayesian Kernel Machine Regression, while adjusted for sex, age, race/ethnicity, educational attainment, poverty income ratio, smoking status, alcohol consumption, physical activity, body mass index, average daily energy intake, family history of diabetes, hypertension, alanine aminotransferase, gamma-glutamyl transferase, and estimated glomerular filtration rate.
